# Determinants of Inapparent and Symptomatic Dengue Infection in a Prospective Study of Primary School Children in Kamphaeng Phet, Thailand

**DOI:** 10.1371/journal.pntd.0000975

**Published:** 2011-03-01

**Authors:** Timothy P. Endy, Kathryn B. Anderson, Ananda Nisalak, In-Kyu Yoon, Sharone Green, Alan L. Rothman, Stephen J. Thomas, Richard G. Jarman, Daniel H. Libraty, Robert V. Gibbons

**Affiliations:** 1 Division of Infectious Disease, State University of New York, Upstate Medical University, Syracuse, New York, United States of America; 2 Department of Epidemiology, Rollins School of Public Health, Emory University, Atlanta, Georgia, United States of America; 3 Department of Virology, United States Army Medical Component, Armed Forces Research Institute of Medical Sciences, Bangkok, Thailand; 4 Center for Infectious Disease and Vaccine Research, University of Massachusetts Medical School, Worcester, Massachusetts, United States of America; University of California, Berkeley, United States of America

## Abstract

**Background:**

Dengue viruses are a major cause of morbidity in tropical and subtropical regions of the world. Inapparent dengue is an important component of the overall burden of dengue infection. It provides a source of infection for mosquito transmission during the course of an epidemic, yet by definition is undetected by health care providers. Previous studies of inapparent or subclinical infection have reported varying ratios of symptomatic to inapparent dengue infection.

**Methodology/Principal Findings:**

In a prospective study of school children in Northern Thailand, we describe the spatial and temporal variation of the symptomatic to inapparent (S:I) dengue illness ratio. Our findings indicate that there is a wide fluctuation in this ratio between and among schools in a given year and within schools over several dengue seasons. The most important determinants of this S:I ratio for a given school were the incidence of dengue infection in a given year and the incidence of infection in the preceding year. We found no association between the S:I ratio and age in our population.

**Conclusions/Significance:**

Our findings point to an important aspect of virus-host interactions at either a population or individual level possibly due to an effect of heterotypic cross-reactive immunity to reduce dengue disease severity. These findings have important implications for future dengue vaccines.

## Author Summary

Dengue viruses are a major cause of illness and hospitalizations in tropical and subtropical regions of the world. Severe dengue illness can cause prolonged hospitalization and in some cases death in both children and adults. The majority of dengue infections however are inapparent, producing little clinical illness. Little is known about the epidemiology or factors that determine the incidence of inapparent infection. We describe in a study of school children in Northern Thailand the changing nature of symptomatic and inapparent dengue infection. We demonstrate that the proportion of inapparent dengue infection varies widely among schools during a year and within schools during subsequent years. Important factors that determine this variation are the amount of dengue infection in a given and previous year. Our findings provide an important insight in the virus-host interaction that determines dengue severity, how severe a dengue epidemic may be in a given year, and important clues on how a dengue vaccine may be effective.

## Introduction

Dengue virus infection can manifest as a clinically inapparent infection, an undifferentiated febrile illness, classic dengue fever (DF), or its more severe form, dengue hemorrhagic fever (DHF) [1]. Inapparent dengue infection is defined as a dengue virus infection that results in no clinical manifestations or an illness that is mild and is not associated with a visit to a health care provider or an absence from school or work due to illness. Inapparent dengue infection because of its nature is not detected by surveillance programs as most programs use visits to a health care provider or hospitalization as an indicator of dengue illness. Thus, inapparent dengue infection represents a burden of dengue infection that goes undetected and hence “inapparent”. The majority of dengue infections in children is thought to be inapparent or present as an undifferentiated febrile illnesses [2], [3]. Determining the incidence of inapparent dengue infection requires detailed prospective cohort studies of populations in dengue endemic areas that can detect dengue infection by paired dengue antibody serology without an associated clinical illness during the time of seroconversion. Few studies of this scope have been performed and thus our knowledge on the full burden of dengue infection is limited. In the first study of this type, a 2-year (1980–1981) school-based study involving 1,757 children, ages 4–16 years, was conducted in Bangkok, Thailand [4]. In this study a symptomatic-to-inapparent (S:I) ratio of 1∶8 was found. A 4-year study was conducted in Managua, Nicaragua in approximately 3,800 schoolchildren, ages 2–9 years, during 2004–2008 [5]. The ratio of S:I infections was 1∶18 during the first year of the study and 1∶3 during the fourth year of the study. A 3-year study, 2000 to 2002, was conducted in 2,536 adults, ages 18–66 years, in West Java, Indonesia [6]. The S:I ratio in this study was 1∶3. Two studies were performed in Kamphaeng Phet, Thailand, Kamphaeng Phet Study I (KPSI) in 1998–2002 and KPSII in 2004–2008. The overall S:I ratio in KPS I was 1∶0.9 and in KPS II an S:I ratio of 1∶3 [7], [8]. We previously reported that there was variation in the S:I ratio during KPS I that varied by school and year with some schools experiencing an S:I ratio of 2∶1 in one year and another school experiencing an S:I ratio of 1∶9 in another year [9].

The pathogenesis of severe dengue disease, DHF, is thought to be a consequence of a heightened immune response due to cross-reactive T-cell responses and/or enhancing dengue antibody during secondary dengue virus infection [10], [11]. It is recognized that dengue infection occurs across the clinical spectrum from subclinical to dengue fever to severe shock and hemorrhage. Understanding the pathogenesis and epidemiology of inapparent dengue infections is important to understand the full burden of dengue infection in a population, its role in underestimating the degree of dengue transmission in a community, and the host and viral factors responsible for influencing subclinical infections such as sequence of serotype infection, heterotypic protective antibody, and host genetic factors. Here we report the spatial and temporal trends of the S:I ratio in dengue infection in a five-year prospective cohort study conducted in Northern Thailand and the epidemiologic factors associated with this variation.

## Materials and Methods

This protocol was reviewed and approved by the Human Use Review and Regulatory Agency of the Office of the Army Surgeon General, the Institutional Review Board of the University of Massachusetts Medical School, and the Thai Ethical Review Board of the Ministry of Public Health, Thailand. All parents/guardians of all children who participated in the study provided informed consent.

The study methods have been previously described [7], [12]. Briefly, this study was conducted in the subdistrict Muang, Kamphaeng Phet Province, Thailand during 1998 to 2002. At the time of the study, subdistrict Muang had 92 public schools of which 12 primary schools were selected to participate in this study based on reliable road access, a desire to participate in the study, and a location within a 3-hour driving radius from the field station laboratory. Children were recruited during January 1998 from grades 1 through 5 and eligible to remain in the study until graduation from 6^th^ grade. During each subsequent year, new 1^st^ grade students were enrolled into the cohort each January. Exclusion criteria included intent to move outside of the study area during the 12 months following enrollment and a history of thalassemia requiring blood transfusion. Baseline demographic information, height and weight, and a blood sample were obtained every January. Evaluations of the entire study population (height, weight, blood sample for dengue serology) occurred three times during the surveillance period (June 1st, August 15th and November 15^th^) of each year. Active acute illness case surveillance of the study participants occurred during the dengue season from June 1^st^ to November 15^th^. Students who were absent from school or visited the school nurse with fever were evaluated the same day with a symptom questionnaire and an oral temperature was obtained. Students with a history of fever in the previous 7 days or an oral temperature ≥38°C were evaluated with a physical exam and an acute illness blood sample was obtained. A convalescent blood sample was obtained 14 days later. Throughout June to November, including weekends and holidays, research nurses tracked children who reported to the public health clinic with an illness or were admitted to the hospital. Students were evaluated using the same methods as above.

The laboratory assays used to detect acute dengue infection were polymerase chain reaction [12], hemagglutination-inhibition assays (HAI) [13], and anti-dengue Immunoglobulin (IgM/IgG enzyme immunoassay (EIA) [14]. Dengue virus isolation in *Toxorhynchites splendens* mosquitoes and enzyme immunoassay were performed for identification of dengue virus serotypes [15], [16], [17], [18].

Clinical definitions of serologically or virologically confirmed dengue virus infection were based on evidence of acute dengue infection and a school-absence associated with fever. By EIA, acute dengue virus infection was defined as a dengue virus-specific IgM level of 40 units or more. Symptomatic dengue virus infection was further classified as symptomatic non-hospitalized or symptomatic hospitalized DF based on their admission into the hospital as decided by the treating physician. Hospitalized symptomatic DF was further defined as either DF or dengue hemorrhagic fever (DHF) using World Health Organization criteria as previously described [7].

Inapparent (subclinical) dengue virus infection was defined as a four-fold rise in HAI antibody against any dengue virus serotype between two sequential sera obtained during the surveillance months (June, August or November) without a febrile illness identified during active surveillance during the time period that seroconversion occurred. For example in sera obtained from a volunteer in June and August where an HAI antibody rise of four-fold was detected to dengue virus with no period of school-absence with fever in the same period was classified as an inapparent dengue infection. Sera were tested concurrently for JEV specific HAI antibody to exclude JEV infection and antibody cross-reactivity as a cause for a fold-four rise in dengue antibody. HAI assays were performed per referenced assays according to standard protocols and interpreted using World Health Organization recognized standards of HAI antibody in acute dengue virus infection [19].

Statistical analyses were performed using SPSS for Windows version 12.0 (SPSS Inc.) and SAS analytic software, version 9.1 (Cary, NC: SAS Institute, Inc.). Only symptomatic and inapparent dengue infections that were detected between June and November each year were included in the analysis. This was done to avoid misclassification of mild, non-hospitalized cases as inapparent infections and altering the SI ratio as an artifact of surveillance. To eliminate bias and due to the small number of primary dengue infections noted in this population, only secondary dengue infections were included for analysis.

Incidence rates were determined using the total study population at the time of surveillance as the denominator. Pearson's chi-square and Spearman correlational analysis were used to identify factors that were significantly associated with the proportional occurrence of symptomatic infection by school and by year. These proportions were then translated into SI ratios using the conversion that the ratio of symptomatic to inapparent (S:I) infections is equal to the probability of being symptomatic over the probability of being inapparent for a given subgroup, i.e.

*Ratio_SI_* = 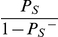 (where P_S_ =  the proportion of infections that were symptomatic). 95% confidence intervals were calculated for the upper and lower limit of the probability of symptomatic infection as 
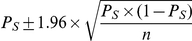
 and then translated to ratios as above. Where the lower confidence interval for the probability of symptomatic infection was negative or the upper limit was greater than or equal to 1, these limits were reset to be 0 and 0.9999 to restrict the range to legitimate values for a ratio.

Logistic regression models were constructed to evaluate the probability a child's infection was inapparent, given school-level characteristics of past (the year prior to a child's infection) and present (the year of the child's infection) dengue epidemics at that school. School-level characteristics that were considered included the incidence of dengue infection, the proportion of infections that were inapparent, the proportion of polymerase chain reaction (PCR)-positive infections that were DENV-1 – DENV-4, and the number of serotypes in circulation (as detected by PCR) at that school. The incidence, proportion inapparent, and the proportions of PCR-positive infections by serotype were categorized into quartiles with the upper limit based upon the observed range for each variable. Statistical analysis accounted for both clustering of children by school and the longitudinal observation of children who may have experienced multiple infections, using SAS PROC GLIMMIX procedure and two levels of random effects. Multiple individual models were constructed, controlling for each of the exposure variables singly and incorporating the random effects.

## Results

### Population Demographics

**Figure 1:**
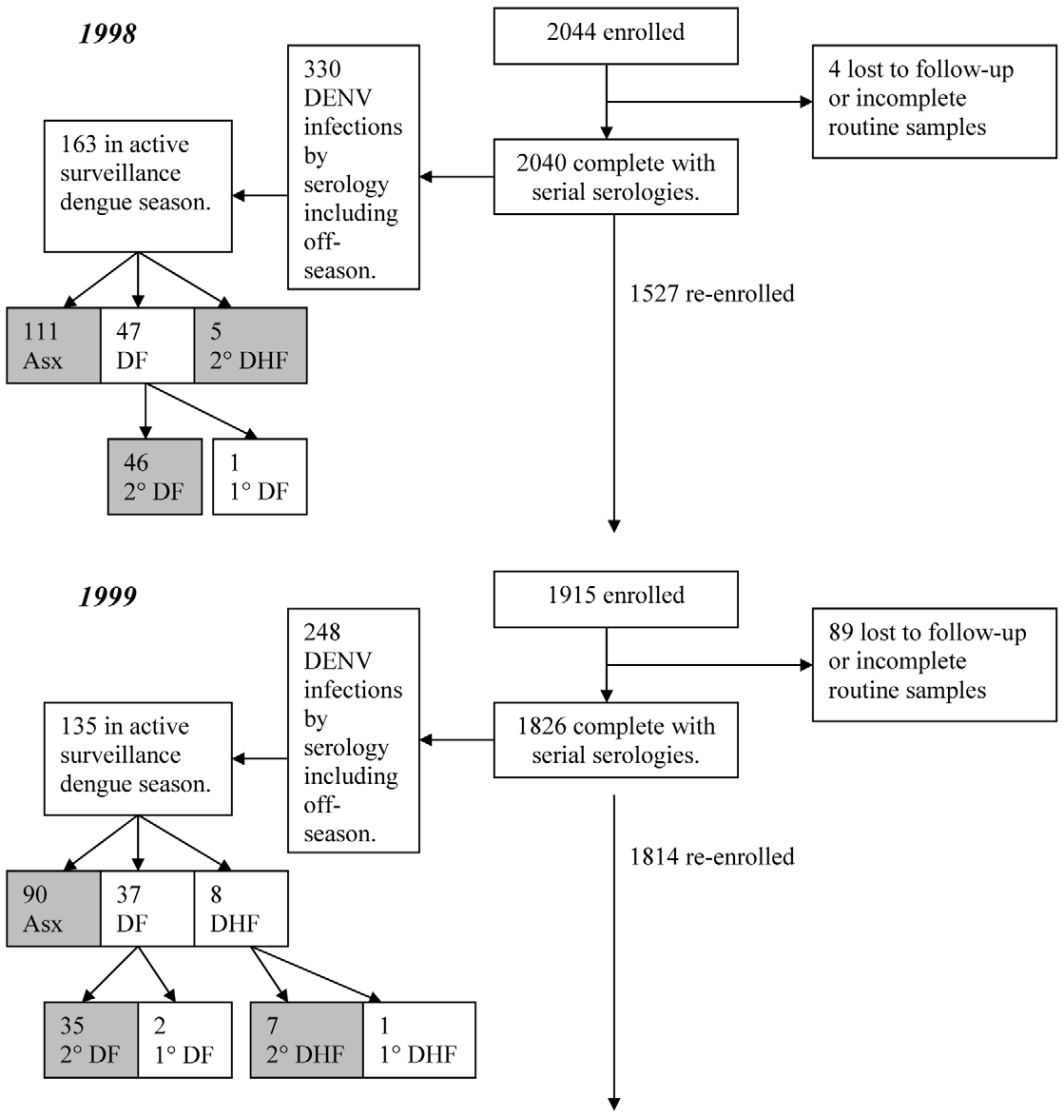
Flow diagram of study population and data analysis. Flow diagram that demonstrates the population enrolled and disease outcomes used in the analysis.

The study population characteristics and incidence of acute dengue infection was previously reported [7], [12]. In January 1998, 2,214 students were initially enrolled in the study with 2,044 remaining for the start of the surveillance. The overall flow of study enrollment and case identification is demonstrated in the flow diagram, figure 1. There was a gradual decline of the study population at the start of active surveillance: 2,044 in 1998; 1,915 in 1999; 2,203 in 2000; 2,011 in 2001; and 1,759 in 2002. The gradual decline of the study population over time is a reflection of the changing demographics of the surveillance schools with smaller 1^st^ grade classes. The mean dropout rate over the study period was 5% primarily due to movement of families out of the surveillance schools. No differences in sex distribution were noted from year to year and between schools (data not shown).

1,024 dengue virus infections were detected in total over the 5 years of the study; 909 children experienced at least one dengue infection during the study period and 115 experienced a second infection. Restricting analyses to the 615 infections detected during the active surveillance period (June 1– November 1), 66% (406) were inapparent and 34% (209) were symptomatic. The median duration of enrollment in the cohort study was two years (25^th^ percentile: 1 year and 75^th^ percentile: 4 years). 3331 children were enrolled for at least one full year. 27.3% of children experienced at least one dengue infection during their period of enrollment. The vast majority of acute infections were secondary by EIA (98%); only 4 infections detected during the active surveillance period were primary.

The one-year incidence of total dengue infection and its constituents, symptomatic and inapparent infection was: for 1998 16.2% (of which 68.1% of those infection detected during the active surveillance period were inapparent); for 1999 13.6% (66.7% inapparent); for 2000 5.2% (81.3% inapparent) for 2001 17.5 (58.0% inapparent); and for 2002 7.1% (66.2% inapparent). There were no fatal cases of dengue. DHF represented 14.8% of symptomatic infections, with the proportion varying yearly from a minimum of 9.6% in 1998 to a maximum of18.2% in 2000. There were no significant differences in the S:I ratio between males and females (p = 0.372 by Pearson chi-square).

### Yearly Variation of the Symptomatic to Inapparent (S:I) Ratio

**Figure 2:**
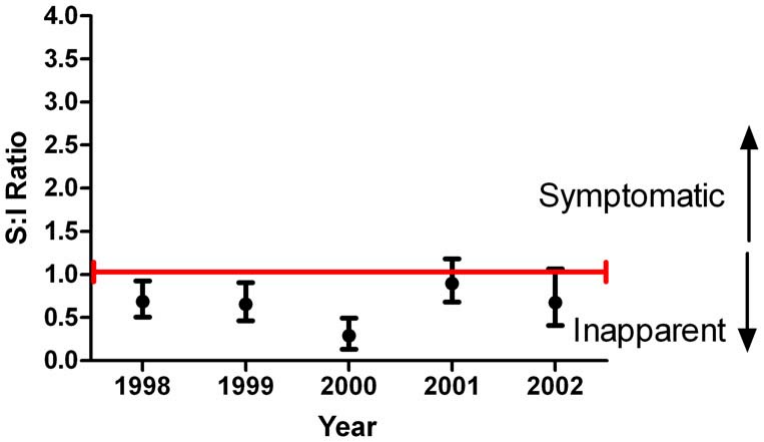
Symptomatic to inapparent ratio for all schools by year. Figure that demonstrates the S:I ratio for all schools in a given year.

Figure 2 demonstrates the annual variation of the S:I ratio for the study population. The S:I ratio in this figure and all figures is transformed into a single, scaled integer which represents the number of symptomatic infections per 1 case of inapparent infection. A value of 1 denotes equal numbers of symptomatic and inapparent cases, an integer greater than 1 indicative of increasing numbers of symptomatic infections, and an integer less than 1 indicative of increasing numbers of inapparent infections. As shown, there is a significant annual variation of this ratio (p<.0001 by Pearson chi-square) with a relatively severe year noted in 2001.

### S:I Ratio and Age

**Figure 3:**
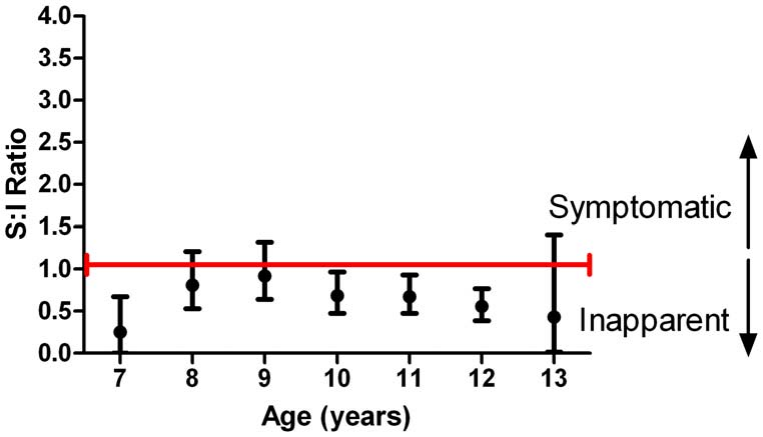
Symptomatic to inapparent ratio by age. Figure that demonstrates the S:I ratio for each age.

As demonstrated in figure 3, there was no correlation between age and the S:I ratio (p>.30 by Pearson chi square, after categorizing into three-year intervals). Further, there was no difference in the proportion of dengue infections that were hospitalized by age (not shown). These findings are contrary to the current belief that more severe dengue is seen among older children and young adults. There were however, four cases detected in the 15 and 16 years age group over the 5 years of the study: all were symptomatic dengue infections.

### Variation of the S:I Ratio within Schools by Year

The spatial diversity of the S:I ratio was examined by schools and by year. Children attending a given school tended to come from the same village, therefore stratifying on school can provide insight into smaller-scale spatial trends in disease and transmission. The total distance across the study site was 30 miles and therefore schools (and neighborhoods) tended to be separated by several miles.

**Figure 4:**
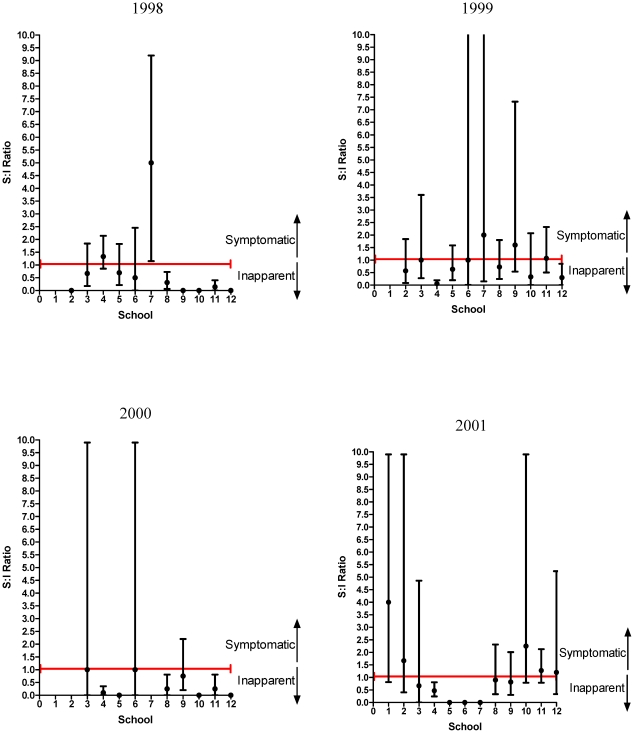
Symptomatic to Inapparent Ratio by Year and School. Figure that demonstrates the temporal and spatial variation of the S:I ratio. Of note the error bars show 95% confidence intervals for the SI ratio. The upper limits that extend to 10 have actual upper limits of 999 (indicating that the upper limit for the probability of symptomatic infection was greater than one) and were therefore adjusted to be 9.9 to make the upper limit estimable. A value of zero indicates that all cases were inapparent in that school. Missing values indicates there were no acute dengue infections for that school and year.

As demonstrated in figure 4, there was considerable diversity in the S:I ratio amongst schools. In 1998, school 7 had much more severe disease with an S:I ratio of 5.0 as compared to other schools during that year. In 1999, schools 6, 7 and 9 were more severe than other schools; in 2000 school 3, 6, and 9 were more severe; in 2001 schools 2, 10 and 12 were more severe and in 2002, schools 4 and 10.

### Variation of the S:I Ratio for Each School over Time

**Figure 5:**
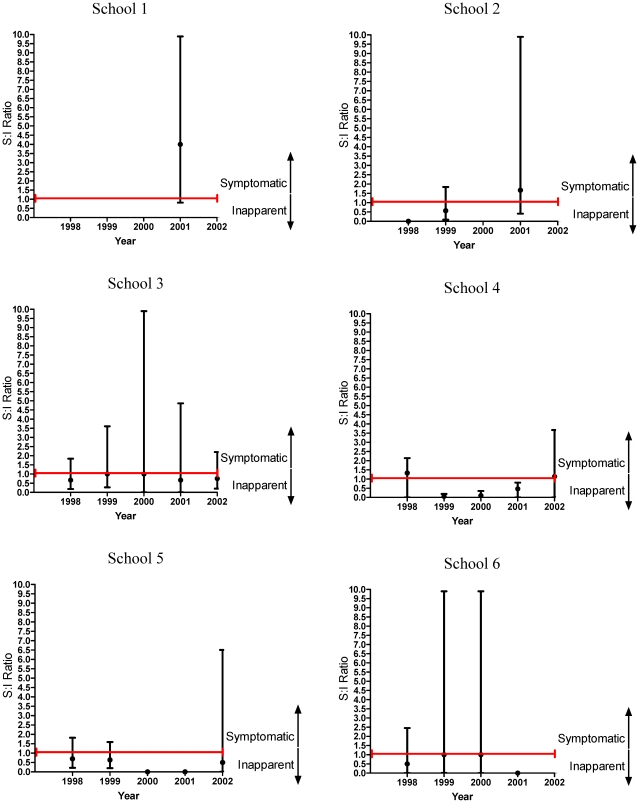
Symptomatic to Inapparent Ratio for Schools 1 to 12 by Year. Figure that demonstrates the individual experience of each school in the yearly variation of the S:I ratio. Similar to figure 4 the error bars show 95% confidence intervals for the SI ratio. The upper limits that extend to 10 have actual upper limits of 999 (indicating that the upper limit for the probability of symptomatic infection was greater than one) and were therefore adjusted to be 9.9 to make the upper limit estimable. A value of zero indicates that all cases were inapparent in that school. Missing values indicates there were no acute dengue infections for that school and year.

Temporal diversity of the S:I ratio was also examined for a given school over time as demonstrated in figure 5. In general each school had a unique experience with regard to changes in the S:I ratio over time. For example, school 8 had relatively mild epidemics every year from 1998–2002, but small-scale annual oscillations in severity were observed. This was different than school 10, which had relatively mild epidemic years from 1998 to 2000, followed by relatively severe years in 2001 and 2002. School 7 had a clinically-severe epidemic in 1998, a less a severe epidemic in 1999, and a mild epidemic in 2001. Though each school had unique characteristics regarding timing and the period of the changing S:I ratios in their population, it is striking that in general, there were cyclical shifts in the S:I ratio with 1 or 2 years of relative inapparent dengue seasons were followed by more severe dengue years.

### Significant Factors Associated with the Spatial and Temporal Change in the S:I Ratio

All four dengue virus serotypes were detected over the study period, though the number of serotypes in circulation varied each year. In 2000, for example, only DENV-2 was detected in PCR+ symptomatic individuals while in 2002, all four DENV serotypes were detected. One predominant dengue serotype was usually the cause of an outbreak in any given year for a specific school as previously described [12].

A higher incidence of infection at a given school, for a given year, was associated with a lower proportion of inapparent infections (i.e., a higher S:I ratio) during that epidemic season (OR = 0.62, 95% CI = 0.53–0.74 for a 25% increase in incidence) (Table 1). Similarly, having a higher number of serotypes in circulation for a given year was associated with a decreased likelihood of inapparent infections (OR = 0.78, 95% CI: 0.62–0.99). Serotype-specific effects were observed, with a higher proportion of DENV-3 circulating at a given school being associated with a lower likelihood of inapparent infections (OR = 0.82, 95% CI: 0.68–0.99) and a higher proportion of DENV-2 being weakly associated with a higher likelihood of inapparent infections (OR = 1.12, 95% CI: (0.98–1.28).

**Table 1:** Associations of the probability of subclinical dengue infection with epidemic characteristics.*

Present year's epidemic characteristics			
	Regression coefficient (beta)	OR for a one unit increase in the variable(95% CI) †	Median(Range) **
Dengue incidence	**−0.470**	**0.62** **(0.53–0.74)**	0.06(0–0.41)
Proportion DENV-1	−0.016	0.98(0.90–1.07)	0(0–1)
Proportion DENV-2	0.111	1.12(0.98–1.28)	0.64(0–1)
Proportion DENV-3	**−0.196**	**0.82** **(0.68–0.99)**	0(0–1)
Proportion DENV-4	0.130	1.14(0.39–3.32)	0(0–1)
Number of DENV serotypes in circulation	**−0.247**	**0.78** **(0.62–0.99)**	1(1–3)

Previous year's epidemic characteristics			
	Regression coefficient (beta)	OR for a one unit increase in the variable(95% CI)	Median(Range)
Previous year's proportion inapparent	−0.161	0.85(0.70–1.03)	0.61(0.20–1)
Previous year's dengue incidence	**0.293**	**1.34** **(1.11–1.61)**	0.09(0–0.41)
Proportion DENV-1	0.049	1.05(0.79–1.40)	0(0–1)
Proportion DENV-2	−0.153	0.86(0.69–1.06)	0.64(0–1)
Proportion DENV-3	0.275	1.32(0.93–1.87)	0(0–1)
Proportion DENV-4	**---**	**---**	---
Number of DENV serotypes in circulation	0.028	1.03(0.67–1.58)	1(1–3)

*Epidemic characteristics at a given school, for a given epidemic year. Performed as individual logistic regression models with subclinical infection as the outcome variable and each epidemic characteristic as a single exposure variable, and incorporating random effects for the individual and the individual's school of attendance.

**. Each exposure variable was aggregated across each school and for each year. The median represents the midpoint of these aggregated values.

**†:** A one-unit increase for proportions (incidence, proportion DENV-1 etc) was defined as a one-quartile increase in value. Quartiles were calculated based upon the range of values (e.g., if incidence had a range of 0–40%, the upper limit for calculating quartiles of incidence was 40%, not 100%). A one-unit increase in the number of serotypes in circulation compared 2 serotypes in circulation to 1 serotype in circulation, for example.

When considering the influence of a previous year's epidemic characteristics on the subsequent epidemic's S:I ratio at a given school, the prior incidence of infection had a strongly positive association with the subsequent S:I ratio. That is, the higher the incidence at a given school the year previous, the greater the probability a child experienced an inapparent infection the following year (OR = 1.34 for a 25% increase in incidence, p<0.01). A higher proportion of symptomatic infections at a given school for the previous epidemic season was weakly associated with an increased likelihood of inapparent infection the following year (OR = 0.85, 95% CI: 0.70–1.03).

**Figure 6:**
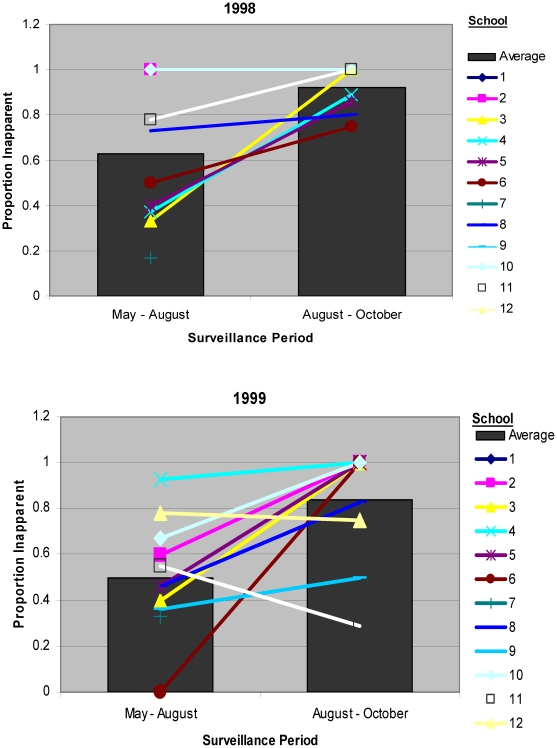
Proportion of infections that were inapparent by school and year. Figure that demonstrates the proportion of inapparent infections during a given dengue virus transmission season.

In 1998, 2001, and 2002, there was a significant overall increase in the proportion of inapparent dengue infections during the second half of the active surveillance period for the entire study population (Aug 16-Nov 1) compared to the first half (Jun 1-Aug 15) (p<0.01 for all, figure 6). For 1999 and 2000, the proportion inapparent remained relatively constant between surveillance periods.

## Discussion

Understanding the full burden of dengue infection and in particular the burden of inapparent dengue infection has important public health implications in understanding virus transmission during an outbreak, the degree of under-reporting during an outbreak year and our understanding on the pathogenesis of dengue illness. Our study is unique from other cohort studies on dengue infection for (1) its long-term follow-up of a well-defined school cohort population; (2) its ability to distinguish the entire spectrum of dengue virus infections and (3) determining spatial and temporal patterns associated with dengue virus transmission. In this report we examined the symptomatic to inapparent ratio as an endpoint and as a correlate for disease severity in a population spatially and over time.

Our results demonstrate that the S:I ratio, unlike in previous reported studies, is not fixed and has wide variation in a population in one specific geographic area and over time. Our data demonstrates that dengue disease severity taken as a whole in a population is an aggregate of multiple experiences of disease severity in sub-populations, with some sub-populations experiencing more severe illness and others less severe dengue infections. In particular, we demonstrated that the previous year's incidence of dengue virus infection influences the subsequent year's dengue disease severity with higher incidence years followed by more clinically-inapparent years. A similar trend has been observed at the national level in Thailand- a cyclical nature in dengue outbreaks and DHF with more severe years followed by milder years [20].

Disease severity observed in our study could be related to a number of factors such as the dengue serotype circulating in the population, viral genetic factors associated with severe disease, and the host's preexisting immunity from a prior dengue virus infection to another serotype leading to antibody enhancement and cross-reactive memory T-cells or conversely cross-protective heterotypic antibody. This would be consistent with previous observations on the pathogenesis of severe dengue illness. Our findings did not demonstrate an association between age and the S:I ratio in our population. This might be due to the narrow age range of our study population, ages 7 to 16 years, but more likely due to the competing nature of other more important factors that influence disease severity, such as the previous year's severity. Our study did not show significance for the proportion of a specific dengue virus serotype and the number of circulating serotypes occurring during an epidemic year or the previous year on the S:I ratio though important factors as demonstrated in previous studies. This may be due to the overwhelming influence of dengue infection in general on disease severity rather than specific dengue serotypes.

Our finding that the greater the incidence of dengue infection of the previous year's epidemic, the milder the subsequent year's disease severity, raises the interesting possibility that herd immunity is an important contributor to disease severity. This could occur either by changing the subsequent year's predominant circulating dengue serotype(s) to a new circulating dengue virus serotype because of protective antibody or by changing the host immune response to experience milder illness. We have previously demonstrated that cross-reactive heterotypic dengue neutralizing antibody does not induce sterile immunity but may influence disease severity on an individual level, at least for some viral serotypes [21]. We believe that what we are observing in the fluctuating nature of the S:I ratio in our population is the influence of this heterotypic cross-reactive antibody at a population level. Based on these findings we postulate that, as a dengue outbreak with a high incidence and disease severity occurs in a given year in a population, there is continued heterotypic herd antibody in a population the following year that doesn't provide protective immunity from infection, but enough cross-protective activity to lower disease severity. This could occur either at the individual or population level. Thus the prior year's S:I ratio and incidence has an inverse relationship on the subsequent year's S:I ratio.

Dengue is a global health concern with no effective vaccine to prevent infection. Understanding the nature of inapparent dengue infection has important public health implications in understanding virus transmission, and determining disease burden during an outbreak. Our findings increase the understanding of this disease and the full burden of infection and point to a role of cross-reactive heterotypic antibody in influencing disease severity the year after an outbreak year in a population. Understanding the factors associated with the development of inapparent dengue infection will increase our understanding on the pathogenesis of this disease and have important implications on how a tetravalent dengue vaccine might influence disease severity in a population.

This project and publication was made possible by NIH Grant P01 AI34533 and the United States Army Medical Research and Materiel Command, Ft Detrick MD. Its contents are solely the responsibility of the authors and do not necessarily represent the official views of the National Institutes of Health or the Department of Defense. The funders had no role in study design, data collection and analysis, decision to publish, or preparation of the manuscript.

## Supporting Information

Checklist S1 STROBE checklist
(0.09 MB DOC)
